# Ethics and the Newspapers

**Published:** 1897-06

**Authors:** 


### EDITORIAL.

The office of the Business Manager of THE JOURNAL is 311 and 312 Fitten Building.

The editors are not responsible for views expressed by contributors.

Articles for publication should reach this office not later than the 15th inst.

Address all communications, and make all remittances payable to THE ATLANTA MEDICAL AND SURGICAL JOURNAL, Atlanta, Ga.

Reprints of articles will be furnished, when desired, at a small cost. Requests for the same should always be made *on the manuscript*.

### ETHICS AND THE NEWSPAPERS.

We call especial attention to a thoughtful and timely letter in another place, bearing the above title, from a well known physician of this State, whose name we are asked to withhold. Physicians generally will indorse the views therein expressed.

The late "charges of unprofessional conduct" which two physicians in Atlanta alleged against three of their brother practitioners "for the good of the profession," and the publicity which the whole matter has attained through some undiscovered source, have given three well-esteemed gentlemen some unfortunate and undesired notoriety, has set in motion considerable medical small-talk, and has furnished some newspapers a suitable opportunity to criticise our code of ethics, to suggest corrections of the same, and otherwise to ventilate their ignorance at our expense. We say it was an unfortunate moment when these two gentlemen, in the sudden irruption of their zeal for ethics, determined to lay violent hands upon these supposed violators of the code.

The facts in this very important case appear to have been as follows: A craniectomy was performed by three physicians upon a microcephalic child, with only vague expectations of accomplishing much good. Three weeks after the operation a newspaper reporter heard of the case, so he says, through an acquaintance by mere chance. He approached one of the surgeons and asked certain questions about the patient, apparently for personal information; the surgeon states that he had no idea the case was to be published, and that when he afterwards learned that it had been written up, he endeavored to have it suppressed, or at least to have names omitted. These statements were corroborated by the reporter. It afterwards developed that the patient's family was the chief source of the reporter's information.

Upon the account as published in a daily paper of Atlanta charges of unprofessional conduct were brought, for aiding and and abetting in the publication, and the charges took the usual course in the Atlanta Society of Medicine. After examining into all the facts and evidence in this remarkable and highly important case, the Executive Committee of the Society found that the surgeon above alluded to had been guilty of an "indiscretion in talking of the case to a newspaper man who had sought him for information; that in justice to him the committee is satisfied that when informed of the use to be made of his information he endeavored to prevent its publication and the use of his name." There was no evidence connecting the two other gentlemen with the published article in any way. So ended this great case of *much-ado-about-nothing.*

The matter was sufficiently important, however, for the Atlanta *Constitution* to utilize it as a *point d'appui* upon which to construct a quasi-satirical editorial upon the Code of Medical Ethics. Happily such lamentable exhibitions of ignorance are not common, at least in the editorial rooms of enlightened modern newspapers. After a labored and overworked simile which compares the code with the heathen Juggernaut, in the midst of which we receive the novel information that the code "was probably invented in the dark ages when physicians were supposed to be magicians," we come to this delightful paragraph:

"We are very sorry that the doctors are unable to look at this so-called 'code of ethics' as a sensible public looks at it. The interior aim of it is so ridiculous that we wonder it has not been banished into the limbo of effete things years ago. If there is a Tom Thumb in the profession, 'ethics' demands that all shall be Tom Thumbs; if a nincompoop, then all must conform to this gauge. A physician cannot be applauded for his skill and success without offending those who have less skill. That is the upshot of the whole matter."

The Code of Medical Ethics, written or unwritten, is accepted by reputable medical men everywhere as a guide in their professional relations with their patients, the public and themselves. It seeks to create and maintain an elevated and dignified *esprit de corps* among physicians, and to keep an ancient, learned and honorable profession unspotted from the ways of commercialism. Since when has such an aim been "ridiculous," and how long has such a thing been "effete"?

But that is not all. Hear the *Constitution's* conclusion of the whole matter:

"The essence and substance of real medical ethics is this: That the invention or discovery of one shall be made available for all. Everything beyond this is ridiculous in the extreme, and it is to be regretted for the sake of all concerned that this sound and benevolent code should be twisted and contorted out of all shape to fit the whims of the foolish and the vanity of the envious."

There you have it. If the code of ethics teaches anything, it is that the "invention or discovery of one shall be made available for all." Therefore one marvels at the dense ignorance contained in the first two lines of the last quotation. The *Constitution's* voluntary contribution to the important but delicate subject of medical ethics is anything but creditable.

We desire to supplement these remarks with the concluding portion of the report of the Committee:

In view of the fact that recent publications in the daily press of this city have given great notoriety to the proceedings of the Atlanta Society of Medicine, and, further, that the "code of medical ethics" which this society professed to maintain has been grossly misrepresented and perverted by such sensational articles, to the manifest impairment of the dignity and influence of our honored profession, your committee respectfully recommends that this report be published in the interests of all concerned. A wise man has well said that "there is nothing so dangerous as ignorance"; therefore, for the enlightenment of those ignorant of the true spirit and intent of the "code of medical ethics," we would state that only the grossest ignorance or most malicious perversion of the truth could possibly distort this wise, beneficent, and moral instrument into "an antiquated engine of torture," "a relic of the dark and barbarous ages," "dragged forth" by "a set of jealous and rusty old fogies" for the "inquisitorial" purpose of "crushing out the progressive spirit, rising genius" and "advanced views" of "enlightened doctors in their skillful efforts to relieve suffering humanity." The medical profession, especially as organized in its various societies, like every other organization, be it a church, a court of law, or any other civic body, has its laws, its regulations and methods of procedure. The common experience of mankind has shown the imperative necessity of such laws and regulations and for such standard of conduct or "ethics," where the beneficent and far-reaching results of united and harmonious organization are sought. The medical profession is no exception to this law. It would indeed be strange if it were otherwise, for surely there is no profession or calling in which the interests of society in general are more vitally and delicately involved! Hence it is we find that the medical profession has a code of laws or "ethics" governing its members in their varied duties and relations to the public and to themselves. This code, as we now have it, is the result of the experience and practical test of many centuries. It is true that our present code, which is that of the American Medical Association, is but little more than forty years of age, yet, looking backward, we find that its essential principles were inculcated in the famous "oath of Hippocrates," the so-called "father of medicine," and was administered to members entering our profession some 357 years before Christ. Our benevolent and noble calling, in its history, indeed, extends so far back into the past that its history, in the proper sense of that term, is lost in traditions amidst the shadows and glooms of a remote antiquity. It was probably coeval with "man's necessity," and in its divine gifts illustrated "God's opportunity." The very heathen, as we term them, of ancient Greece and Rome claimed our origin from one of their gods, Apollo, yet as "the thoughts or men are broadened with the process of the times," even so has our loved profession advanced with the enlightened "spirit of the age." Emerging from darkness, it has seen the dawn of a better day, and has endeavored by its principles incorporated in its "code of ethics" to illustrate the teachings of "the Great Physician," "Whatsoever ye would that men should do to you, do ye even so to them." In enforcing its mandates and demanding allegiance to its principles, this code does but claim "that which is lawful and right." Liberal and true, the "code of medical ethics" has been established by this profession, which has been compelled to recognize that as in the case of the church, yea, even of "the twelve" who followed the Christ, there are those unworthy of their high calling. From earliest times there have been those who, under the name of physicians, prey upon the necessity and credulity of mankind. Our code, so far from "repressing discovery," or "retarding" the perfecting of its art, encourages and even demands that each of its members shall contribute of his talents, be it one or five, to the advancement of all, and the good of mankind. Through our medical societies and medical periodicals such discoveries and improvements are made known; are tested, and accepted or rejected, and that after careful and wise investigation; they are not paraded to an uninstructed and credulous public through the medium of paid or other advertisements in the secular press. Such is the genius of our code, standing pure and uncontaminated amidst certain influences of this age, which tend to lower our standard of professional honor, she reaches out her hands and cries, "Entreat me not to leave thee!" We compel no man to join our society, but to those who do we say, "You shall not for mere unscrupulous private gain and personal advertisement, regardless of the means employed, appropriate the cloak of an honorable profession!"—*J. C. Olmsted, M.D., W. S. Kendrick, M.D., and J. S. Todd, M.D., Committee.*

---

ONE of the interesting features attending the late Congress of American Physicians and Surgeons in Washington was the unveiling of the statue of the late Professor S. D. Gross, in the grounds of the Smithsonian Institution. The presentation address was delivered by Dr. C. H. Mastin, of Mobile, and the statue was accepted on behalf of the Government by Surgeon-General George M. Sternberg. Dr. W. W. Keen, of Philadelphia, delivered an address upon the life, work and character of Dr. Gross.

